# Acoustic Cry Characteristics in Preterm Infants and Developmental and Behavioral Outcomes at 2 Years of Age

**DOI:** 10.1001/jamanetworkopen.2022.54151

**Published:** 2023-02-01

**Authors:** Andrew W. Manigault, Stephen J. Sheinkopf, Brian S. Carter, Jennifer Check, Jennifer Helderman, Julie A. Hofheimer, Elisabeth C. McGowan, Charles R. Neal, Michael O’Shea, Steven Pastyrnak, Lynne M. Smith, Todd M. Everson, Carmen J. Marsit, Lynne M. Dansereau, Sheri A. DellaGrotta, Barry M. Lester

**Affiliations:** 1 Brown Center for the Study of Children at Risk, Women and Infants Hospital of Rhode Island, Providence; 2 Thompson Center for Autism and Neurodevelopmental Disorders, University of Missouri, Columbia; 3 Children’s Mercy Hospital, Kansas City, Missouri; 4 Wake Forest School of Medicine, Winston-Salem, North Carolina; 5 University of North Carolina Chapel Hill School of Medicine, Chapel Hill; 6 Departments of Psychiatry and Pediatrics, Alpert Medical School of Brown University, Providence, Rhode Island; 7 University of Hawaii John A Burns School of Medicine, Honolulu, Hawaii; 8 Spectrum Health-Helen Devos Hospital, Grand Rapids, Michigan; 9 Harbor-University of California, Los Angeles Medical Center, Torrance; 10 Rollins School of Public Health, Emory University, Atlanta, Georgia

## Abstract

**Importance:**

Acoustic cry characteristics have been associated with severe medical problems in newborns. However, little is known about the utility of neonatal acoustic cry characteristics in the prediction of long-term outcomes of very preterm infants.

**Objectives:**

To evaluate whether acoustic characteristics of infant cry at neonatal intensive care unit (NICU) discharge are associated with behavioral and developmental outcomes at age 2 years in infants born very preterm.

**Design, Setting, and Participants:**

Infants born less than 30 weeks postmenstrual age (PMA) were enrolled from April 2014 through June 2016 as part of a multicenter (9 US university affiliated NICUs) cohort study and followed to adjusted age 2 years. Reported analyses began on September 2021. Data were analyzed from September 2021 to September 2022.

**Exposures:**

The primary exposure was premature birth (<30 weeks PMA).

**Main Outcomes and Measures:**

Cries were recorded during a neurobehavioral examination administered during the week of NICU discharge. Cry episodes were analyzed using a previously published computerized system to characterize cry acoustics. Year-2 outcomes included the Bayley-III Composite scores, Child Behavior Checklist (CBCL) and the Modified Checklist for Autism in Toddlers (M-CHAT R/F), dichotomized using clinically significant cutoffs (<85 on Bayley Language, Cognitive and/or Motor Composite scores, T-score >63 on the CBCL Internalizing, Externalizing and/or Total Problem Scales and total M-CHAT R/F score >2).

**Results:**

Analyzed infants (363 participants) were primarily male (202 participants [55.65%]) and had a mean [SD] gestational age of 27.08 [1.95] weeks). Cross-validated random forest models revealed that cry acoustics were associated with 2-year outcomes. Tests of diagnostic odds ratios (DOR) revealed that infants who exhibited total problem behavior CBCL scores greater than 63 at age 2 years were 3.3 times more likely (95% CI, 1.44-7.49) to be identified as so by random forest model estimates relative to other infants (scores ≤63); this association was robust to adjustment for family-wise type-I error rates and covariate measures. Similar associations were observed for internalizing (DOR, 2.39; 95% CI, 1.04-5.47) and externalizing (DOR, 2.25; 95% CI, 1.12-4.54) scores on the CBCL, clinically significant language (DOR, 1.71; 95% CI, 1.10-2.67) and cognitive (DOR, 1.70; 95% CI, 1.00-2.88) scores on the Bayley-III, and a positive autism screen on the M-CHAT (DOR, 1.91; 95% CI, 1.05-3.44).

**Conclusions and Relevance:**

In this cohort study of preterm infants, findings pointed to the potential use of acoustic cry characteristics in the early identification of risk for long-term developmental and behavioral deficits.

## Key Points

**Question:**

Are acoustic cry characteristics in preterm neonates associated with developmental and behavioral outcomes at 2 years of age?

**Findings:**

In this cohort study of 363 preterm infants, acoustic cry characteristics were associated with clinically significant language and cognitive deficits, behavior problems, and a positive autism screen at age 2 years.

**Meaning:**

These findings point to the potential use of acoustic cry characteristics in the early identification of preterm infants that are most at risk for longer term developmental and behavioral deficits.

## Teaser

This cohort study evaluates the association between acoustic cry characteristics in preterm infants and developmental and behavioral outcomes at 2 years of age.

## Introduction

Improvements in medical care have increased the survival rate of infants born less than 30 weeks’ postmenstrual age (PMA); however, these infants remain at high risk of developing motor, cognitive and language delays, and behavior problems.^1,2,3,4,5,6,7,8,9,10,11,12^ Yet not all children born preterm will exhibit these delays and problems, and so tools or biomarkers that can identify the highest risk population are needed to develop targeted supportive services.

The possibility that acoustic characteristics of the infant’s cry could be associated with prematurity is suggested by the findings that medical conditions affecting the brain, such as postasphyxia encephalopathy, meningitis, and *cri du chat* have been associated with “abnormal” cries.^13^ This association led to the hypothesis that cry characteristics could be a measure of the integrity of the central nervous system and be useful in populations with less severe medical problems such as preterm infants.

The paradox in infant cry research is that early technologies to measure acoustic features of the infant’s cry used crude instrumentation; initially the sound spectrograph, which could only measure a few cry features, was used. Cry pitch became the harbinger of pathology because it is audible and high pitch is common in infants with medical problems.^13^ However, with the advent of high-speed computer technology, the sheer amount of cry data that became available was unmanageable for traditional statistical models. The present study brings together a cry analysis system that leverages state-of-the-art signal processing with machine learning technology to take advantage of large and complex data sets to study associations with varied cry acoustic features including energy (ie, loudness), pitch (ie, fundamental frequency), formants (ie, resonance), voicing (ie, vocal fold vibration), and fricatives (ie, vocal tract constriction). We studied preterm infant cries recorded before discharge from the neonatal intensive care unit (NICU) and developmental outcomes at age 2 years and hypothesized that acoustic cry characteristics were associated with neurobehavioral and behavioral deficits.

## Methods

### Study Design

Infants were enrolled in the multicenter, observational Neurobehavior and Outcomes in Very preterm Infants (NOVI) study from April 2014 through June 2016 at 9 US university-affiliated NICUs. Parents of eligible infants were invited to participate at 31 to 32 weeks’ PMA, or when the attending neonatologist determined that survival to discharge was likely. Enrollment and consent procedures were approved by local institutional review boards. All mothers provided written informed consent. Sample size was determined by an a priori power analysis. This cohort study adhered to the Strengthening the Reporting of Observational Studies in Epidemiology (STROBE) reporting guideline.

### Inclusion/Exclusion Criteria

Inclusion criteria were: (1) birth at less than 30 weeks postmenstrual age, (2) parental ability to read and speak English or Spanish, and (3) residence within 3 hours of the NICU and Follow-Up clinic. Exclusion criteria included maternal cognitive impairment (inability to provide informed consent), maternal age of less than 18 years, maternal or infant death, and infants with major congenital anomalies.

### Measures

Cries of the preterm infants were recorded during administration of the NICU Network Neurobehavioral Scale (NNNS)^7^ during the week of NICU discharge (± 3 days). The cries were elicited by handling the baby in a standardized format that is designed to increase the babies’ level of arousal. The stimuli are akin to cries that are elicited during routine caregiving such as a diaper change. Audio recordings were made using an Olympus direct to PCM digital voice recorder and saved in an uncompressed .wav audio format (recording parameters: 16 bit, 48 kHz). Recorders were attached to the side of the infant’s crib at a standardized location and oriented toward the infant’s mouth. This allowed for a standard distance between the infant’s mouth and the recorder during the NNNS examination (approximately 8-9 inches from the infant’s mouth). Episodes of cry vocalizations were identified from these audio recordings. Cry episodes suitable for acoustic analysis were identified based on the absence of background noises that would interfere with the analysis (ie, adult talk, medical equipment noises, and other environmental noises). The identification of usable cries was based on reliability training from a previous study^14^ in which 89% agreement was established in identifying cries appropriate for acoustic analysis. The first suitable cry episode from each examination was excerpted into an uncompressed .wav file for subsequent acoustic analysis. The extracted recordings only contained infant cries and were stripped of any identifiable information. Of the infants with 2-year outcome data, 428 had extractable cries (infant cried during the audio recording, good sound quality, no background noise).

Several acoustic features were used to characterize cry episodes, including energy, fundamental frequency, formants, utterances, voicing, fricatives, and signal quality. Briefly, energy measures sound pressure levels in decibels (which we hear as loudness) as air is expelled from the lungs. Related are utterances which can be long (>500 ms) or short (<500 ms). At the larynx, vibration of the vocal folds produces sound waves including the fundamental frequency (which we hear as pitch). This initial sound is then modified or filtered by the vocal track producing formants. Formants are characteristic features of the resonances of the space, (ie, the vocal tract). For example, a “C” note on a saxophone sounds different than a “C” note on a piano because the resonating chambers are different. Signal quality measures the reliability with which the cry analyzer can measure the acoustic characteristics. Voicing refers to sounds produced by vocal fold vibration. Sounds can also be unvoiced when the vocal folds are not involved but the sound is altered as, for example, with the tongue or lips. Fricatives characterizes how the sound is altered by friction due to constriction of the vocal tract.

Procedures and criteria for collecting maternal and infant variables have been previously described, including cranial ultrasonography readings, interpreted by centralized study neuroradiologists.^13^ Infant medical risk was measured using an index,^15^ in which scores are computed as the sum of dummy variables indicating the presence of 4 morbidities ascertained prospectively^16^ from medical record reviews using Vermont-Oxford Network definitions^17^ of brain injury (from consensus central readings of cranial ultrasounds [that defined periventricular leukomalacia, moderate-severe ventriculomegaly, or parenchymal echodensity]), bronchopulmonary dysplasia, severe retinopathy of prematurity, and necrotizing enterocolitis and/or culture positive sepsis.^15^ Race and ethnicity data (American Indian/Alaska Native, Asian, Black or African American, Native Hawaiian or other Pacific Islander, more than 1 race, unknown or not reported, Hispanic ethnicity) were obtained from maternal interview self-reports (preferred source), or medical record abstraction. We included race and ethnicity as part of standard, unbiased demographic information.

Main outcome measures were completed at 2 years adjusted age (± 2 months). Assessments included the Bayley-III, administered by experienced examiners who were blinded to infant medical conditions in the NICU, and trained by certified Bayley-III trainers to reliability using standardized Bayley-III training protocols.^18^ Parents completed the Child Behavior Checklist (CBCL)^19^ problem scales and the Modified Checklist for Autism in Toddlers (M-CHAT R/F).^20^ These measures were dichotomized using clinically significant cutoffs (<85 on Bayley Language, Cognitive and/or Motor Composite scores, T-score >63 on the CBCL Internalizing, Externalizing and/or Total Problem Scales, and total M-CHAT R/F score >2).^19,20,21^

### Statistical Analyses

As shown in the Figure, digital sound files of cry episodes were subjected to an established, computerized cry analysis system.^18^ Analysis proceeded in 2 phases. The first phase applied a cepstral-based acoustic analysis to extract acoustic parameters with a 12.5 ms frame advance. The second phase organized and summarized this information into cry utterances. The mean (SD) amount of crying analyzed per infant was 17.37 seconds (15.54). Long cry utterances were defined as a cry during the expiratory phase of respiration lasting at least 0.5 seconds. Short cry utterances were less than 0.5 seconds; short and long utterances show distinct acoustic characteristics and thus were analyzed separately. The automated cry analyzer produced 56 acoustic characteristics per cry utterance. Acoustic characteristics for a total of 4431 long (≥500 ms) and 10 270 short (<500 ms) utterances were processed, including a mean (SD) of 12.2 (12.2) and median (IQR) of 9 (6-14) long utterances and a mean (SD) of 28.3 (25.8) and median (IQR) of 20 (13-33) short utterances per child on average. For each acoustic characteristic, we computed infant-level means and infant-level signal quality (ie, the percentages of utterances that produced a valid value for a given acoustic parameter). Infant-level counts of cry utterances were also computed (thereafter referenced as “utterances”). Next, the resulting set of candidate features was reduced, such that features with high rates (>40%) of missing data, near 0 variability, and high (*r* = 0.75) intercorrelation were removed, consistent with published guidelines for machine learning feature filtering.^22^

**Figure.**
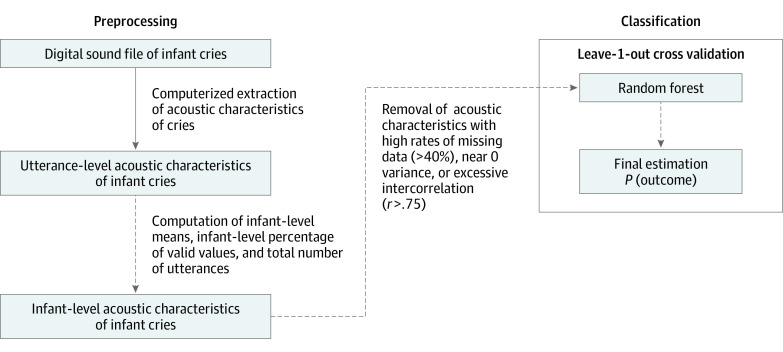
Data Reduction Strategy Flowchart of data reduction strategy whereby infant level estimates for year 2 outcomes were systematically generated using digital sound files of infant cries.

A cross-validated supervised machine learning model (ie, random forest) was used to estimate developmental outcomes at age 2 using all available cry acoustics variables. Random forests were fitted using the ranger R^23^ package on R 4.2 (R Project for Statistical Computing)^24^ using default parameters with exception of number of trees (10 000). Model estimates were evaluated using leave-1-out cross validation, such that the test-train split is repeated for every infant in the data set, random forest models are trained on data containing all except 1 infant, and the held-out infant is used to evaluate whether model estimates generalize to new data. Predicted probabilities generated using leave-1-out cross validation were used to create a receiver operating characteristic (ROC) curve, and pick an optimal probability cut point (based on the shortest distance to perfect prediction).^25^ The resulting binary estimates were used to compute diagnostic odds ratios (DOR),^26^ 95% confidence intervals and *P* values (α = .05 statistical significance threshold). Area under the receiver operating curve estimates were also reported.^26^

A mixed effect binary logistic regression model was fitted (using glmer from the lme4 package^27^) to account for nesting of infants (level 1 unit) within study sites (level 2 unit). Consistent with prior work,^28,29^ the effect of confounding variables was adjusted using a stepwise method, where all covariate measures (ie, sex, low maternal SES, low maternal education, minority race or ethnicity, maternal primary language, no partner at birth, infant medical risk index score, and post menstrual age at birth) were entered as level 1 fixed effects in the first step and the final estimate of our machine learning models was entered as a level 1 fixed effect in the second step. A (nonparametric) likelihood ratio permutation test was used to evaluate whether adding the final machine learning estimate to a model containing covariate measures led to a significant reduction in model deviance. The false discovery rate correction was used to maintain acceptable (α = .05) family-wise type I error rates. Data were analyzed from September 2021 to September 2022.

## Results

The study enrolled 704 infants, of which 556 completed the 2-year assessment (mean [SD] follow-up, 2.29 [0.16] years). Of these, 363 completed the year 2 follow-up, had complete covariate data and valid cry data. Maternal and infant descriptive data of the final sample are shown in Table 1. Comparing participants included (363) vs excluded (341) from final analyses on study variables (ie, all variables listed in Table 1) revealed that excluded infants showed relatively higher rates of motor developmental deficits; χ^2^_1_(541) = 6.25, *P* = .01, OR = 1.77; all other tests were nonsignificant.

**Table 1.** Demographic and Clinical Characteristics of 327 Mothers and 363 Infants Included in the Sample

Variable	No. (%)
Maternal prenatal characteristics	
Maternal age, y, mean (SD) (n = 324)	29.04 (6.12)
Single/no partner at birth	86 (26.30)
Low SES (lowest SES category on Hollingshead scale)	29 (8.87)
Education (<high school)	44 (13.46)
Race	
American Indian/Alaska Native	1 (0.31)
Asian	15 (4.59)
Black or African American	62 (18.96)
Native Hawaiian or other Pacific Islander	2 (0.61)
White	151 (46.18)
More than 1 race	74 (22.63)
Unknown or not reported	22 (6.73)
Ethnicity (Hispanic)	68 (20.80)
Infant characteristics	
Postmenstrual age, mean (SD), wk	
At birth	27.08 (1.95)
At discharge	40.18 (4.85)
Birthweight, g (n = 362)	965.56 (278.76)
Sex	
Female	161 (44.35)
Male	202 (55.65)
Intrauterine growth restriction (<10th percentile)	26 (7.20)
Infant medical risk index score	
Overall, mean (SD)	0.83 (0.85)
Severe brain injury (PVL, VD, PED)	48 (13.22)
Bronchopulmonary dysplasia	173 (47.66)
Severe retinopathy of prematurity	19 (5.23)
Necrotizing enterocolitis/sepsis	62 (17.31)
Problem behavior (score >63 on Child Behavior Checklist)	
Total	35 (9.80)
Internalizing	27 (7.54)
Externalizing	39 (10.92)
Composite (Bayley-III score <85)	
Language	127 (36.81)
Cognitive	76 (22.03)
Motor	56 (16.23)
Positive autism screen on Modified Checklist for Autism in Toddlers	55 (15.28)

Abbreviations: PED, parenchymal echodensity; PVL, periventricular leukomalacia; VD, ventricular dilatation.

Model estimates of 2-year outcomes showed improvements over chance (total CBCL area under ROC [AUROC], 0.619; 95% CI, 0.531-0.707; internalizing CBCL AUROC, 0.540; 95% CI, 0.432-0.647; externalizing CBCL AUROC, 0.627; 95% CI, 0.537-0.717; Bayley-III language AUROC, 0.556; 95% CI, 0.492-0.619; Bayley-III cognitive AUROC, 0.576; 95% CI, 0.502-0.649; Bayley-III motor AUROC, 0.546; 95% CI, 0.467-0.625; Autism screener AUROC, 0.574; 95% CI, 0.493-0.654). Test of DOR (Table 2) implied a statistically significant association between model estimates and observed outcome for total problem behavior scores (OR, 3.29; 95% CI, 1.44-7.49; *P* = .04) that was robust to false discovery rate correction and adjustment for covariate measures. Similar, but nonsignificant tests of DOR were observed for other outcomes, including internalizing problem behavior (OR, 2.39; 95% CI, 1.04-5.47; *P* = .056), externalizing problem behavior (OR, 2.25; 95% CI, 1.12-4.54; *P* = .054), language deficit (OR, 1.71; 95% CI, 1.10-2.67; *P* = .054), cognitive deficit (OR, 1.70; 95% CI, 1.00-2.88; *P* = .054) and positive autism screen (OR, 1.91; 95% CI, 1.05-3.44; *P* = .056). As shown in Table 2, controlling for covariate measures minimally impacted DORs and *P* values. Finally, permuted likelihood ratio tests were significant only for model comparisons evaluating estimates of total problem behaviors.

**Table 2.** Characterizing the Association Between 2-Year Outcomes and Estimates of Machine Learning Models^a^

Outcome	Unadjusted		Adjusted for Covariates		LRT, *P* value^b^
	OR (95% CI)	*P* value	OR (95% CI)	*P* value	
Child Behavior Checklist					
Problem behavior (>63)					
Total	3.29 (1.44-7.49)	.04	3.67 (1.51-8.88)	.03	.01
Internalizing	2.39 (1.04-5.47)	.056	2.39 (0.98-5.83)	.08	.08
Externalizing	2.25 (1.12-4.54)	.054	2.27 (1.08-4.77)	.07	.08
Bayley-III					
Composite (<85)					
Language	1.71 (1.10-2.67)	.054	1.58 (0.98-2.54)	.08	.08
Cognitive	1.70 (1.00-2.88)	.06	2.05 (1.15-3.68)	.056	.07
Motor	1.51 (0.84-2.69)	.17	1.40 (0.72-2.71)	.32	.31
Modified Checklist for Autism in Toddlers					
Positive autism screen	1.91 (1.05-3.44)	.056	1.80 (0.92-3.54)	.10	.10

Abbreviation: OR, odds ratio.

^a^ False discovery rate adjusted *P* values are shown.

^b^ Permuted Likelihood Ratio Tests (LRT) comparing models containing only covariate measures vs covariates and machine learning estimates. Covariate measures included sex, low maternal SES, low maternal education, minority race or ethnicity, maternal primary language, no partner at birth, infant medical risk index score, and post menstrual age at birth.

Examining the top 10 most important variables within each model (see eTable in Supplement 1) revealed that final models used 140 acoustic features across 14 separate models that may be summarized into 7 groups of variables (ie, energy, fundamental frequency, formants, utterances, voicing, fricatives, and signal quality). Of the 7 variable groups, 3 (energy, formants and signal quality) were used at least 20 times, 1 (fundamental frequency) was used 18 times and the remaining 3 (utterances, voicing, and frication) were used 8 times or less.

## Discussion

Using a machine learning (random forest) approach along with contemporary signal processing methods, we have shown that acoustic cry characteristics are associated with some 2-year developmental and behavioral deficits in very preterm infants. Specifically, estimates of models trained using acoustic cry characteristics were associated with clinically significant total problem behavior scores, even after controlling for covariate measures or family-wise type-I error rates. Likelihood-ratio tests also indicated that adding estimates of random forest models trained on cry acoustics to a mixed model containing covariates significantly improved model fit, suggesting a unique association of acoustic cry characteristics with prediction of clinically significant total problem behavior scores at age 2. Similar (but nonsignificant) associations were observed for clinically significant language and motor composite scores on the Bayley-III, internalizing and externalizing scores on the CBCL, and a positive autism screen on the M-CHAT.

Previous work describing abnormal cry acoustics in infants with severe medical problems suggested that cry characteristics might be of diagnostic value in at-risk infant populations where developmental outcome is less clear. Cry acoustics have been associated with prematurity^30,31,32^ and other at risk populations^13^ in the neonatal period but not to longer term developmental or behavioral deficits and typically researchers examined a limited subset of acoustic characteristics. Our findings point to the potential use of acoustic cry characteristics in the early (before NICU discharge) identification of which preterm infants are most at risk for longer term developmental and behavioral deficits and by perhaps to other at-risk and not at-risk populations.

The CBCL findings are noteworthy from a linguistic perspective. Human speech is divided into linguistic and paralinguistic components. The linguistic component refers to words and their syntax. The paralinguistic component is the prosodic features of pitch, loudness, melody, and intonation that convey the affective side of speech. Crying includes only prosodic features that are aligned with the behavioral and emotional problems measured by the CBCL. The effects that we found were on the internalizing problem score which includes anxious/depressed, withdrawn-depressed, and somatic complaint scales and the externalizing problem score that combines the rule-breaking and aggressive behavioral scales. This connection between cry as prosody and later behavioral and emotional problems could lead to the study of neural pathways related to the origins of mental health disorders.

### Limitations

Our study had limitations. One limitation of this work is that we were only able to use cries from 363 of the 556 infants; however, there was only one significant difference between those included and those excluded. This suggests that we need to make improvements in cry collection and extraction. A second limitation is that this is a data driven approach and although the infants were drawn from 9 NICUs, this was still a single cohort study. The next step would be a hypothesis-testing study to determine if these acoustic cry models replicate in other cohorts. Additionally, although acoustic cry analysis may help identify which preterm infants are most at risk for adverse outcomes, these measures do not readily translate into specific intervention strategies.

## Conclusions

Despite its limitations, this work raises the possibility that cry analysis could be developed into a bedside, accessible, and non–labor-intensive machine-interpreted diagnostic similar to electrocardiographic readings. The ability to identify which infants are at highest risk for developmental and behavioral disorders could lead to the development of interventions to mitigate adverse outcomes.

## Notes

Supplement 1. **eTable. **Count of the Top 10 Most Important Variables Broken Down by Model Outcome, Utterance Type and Acoustic Variable Type **eFigure 1. **Feature Importance Estimates From Models Predicting CBCL Composite Scores Using Cry Acoustic Characteristics (Only the Top 10 Features are Shown) **eFigure 2. **Feature Importance Estimates From Models Predicting Bayley-III Composite Scores Using Cry Acoustic Characteristics (Only the Top 10 Features are Shown) **eFigure 3. **Feature Importance Estimates From Models Predicting MCHAT Positive Autism Screen Using Cry Acoustic Characteristics (Only the Top 10 Features are Shown)

Supplement 2.
Data Sharing Statement
