# Supplementary material for: Acoustic Cry Characteristics in Preterm Infants and Developmental and Behavioral Outcomes at 2 Years of Age
Source: JAMA Netw Open. 2023 Feb 1;6(2):e2254151. doi: 10.1001/jamanetworkopen.2022.54151 (PMC9892956; doi:10.1001/jamanetworkopen.2022.54151)
Supplement: Supplement 1. — eTable. Count of the Top 10 Most Important Variables Broken Down by Model Outcome, Utterance Type and Acoustic Variable Type eFigure 1. Feature Importance Estimates From Models Predicting CBCL Composite Scores Using Cry Acoustic Characteristics (Only the Top 10 Features are Shown) eFigure 2. Feature Importance Estimates From Models Predicting Bayley-III Composite Scores Using Cry Acoustic Characteristics (Only the Top 10 Features are Shown) eFigure 3. Feature Importance Estimates From Models Predicting MCHAT Positive Autism Screen Using Cry Acoustic Characteristics (Only the Top 10 Features are Shown) [file jamanetwopen-e2254151-s001.pdf]

Supplemental Online Content

Manigault AW, Sheinkopf SJ, Carter BS, et al. Acoustic cry characteristics in preterm infants and developmental and behavioral outcomes at 2 years of age. *JAMA Netw Open*. 2023;6(2):e2254151. doi:10.1001/jamanetworkopen.2022.54151

eTable. Count of the Top 10 Most Important Variables Broken Down by Model Outcome, Utterance Type and Acoustic Variable Type

eFigure 1. Feature Importance Estimates From Models Predicting CBCL Composite Scores Using Cry Acoustic Characteristics (Only the Top 10 Features are Shown)

eFigure 2. Feature Importance Estimates From Models Predicting Bayley-III Composite Scores Using Cry Acoustic Characteristics (Only the Top 10 Features are Shown)

eFigure 3. Feature Importance Estimates From Models Predicting MCHAT Positive Autism Screen Using Cry Acoustic Characteristics (Only the Top 10 Features are Shown)

This supplemental material has been provided by the authors to give readers additional information about their work.

© 2023 Manigault AW et al. *JAMA Network Open.*

eTable. Count of the Top 10 Most Important Variables Broken Down by Model Outcome, Utterance Type and Acoustic Variable Type

	Total	Energy	Fundamental Frequency	Formants	Utterance	Voicing	Fricatives	Signal Quality
CBCL								
Total Problem Behavior (>63) - long	10	3	1	1	2	1	1	1
Total Problem Behavior (>63) - short	10	1	0	3	0	1	0	5
Externalizing Problem Behavior (>63) - long	10	3	3	1	1	1	1	0
Externalizing Problem Behavior (>63) - short	10	2	0	3	0	1	0	4
Internalizing Problem Behavior (>63) - long	10	3	1	3	0	1	1	1
Internalizing Problem Behavior (>63) - short	10	1	1	4	0	0	0	4
Bayley-III								
Language Composite (<85) - long	10	6	1	0	2	0	0	1
Language Composite (<85) - short	10	3	2	3	0	0	0	2
Cognitive Composite (<85) - long	10	5	2	2	0	0	0	1
Cognitive Composite (<85) - short	10	3	3	1	0	0	0	3
Motor Composite (<85) - long	10	3	3	1	1	1	1	0
Motor Composite (<85) - short	10	3	0	3	0	0	0	4
M-CHAT								
Positive Autism Screen - long	10	5	1	1	1	1	0	1
Positive Autism Screen - short	10	3	0	4	1	1	0	1
Total	140	44	18	30	8	8	4	28

© 2023 Manigault AW et al. *JAMA Network Open.*

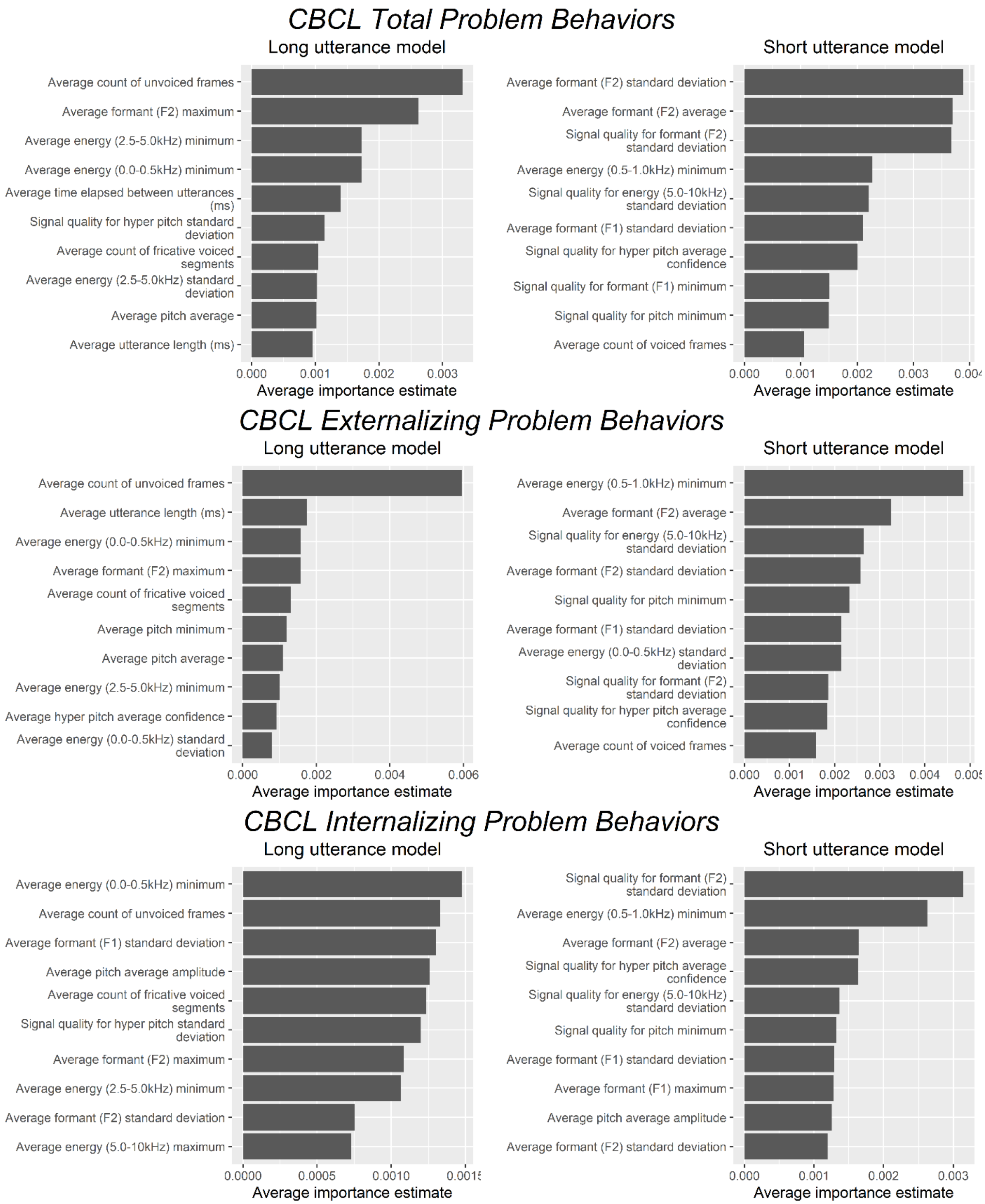

eFigure 1. Feature Importance Estimates From Models Predicting CBCL Composite Scores Using Cry Acoustic Characteristics (Only the Top 10 Features are Shown)

© 2023 Manigault AW et al. *JAMA Network Open.*

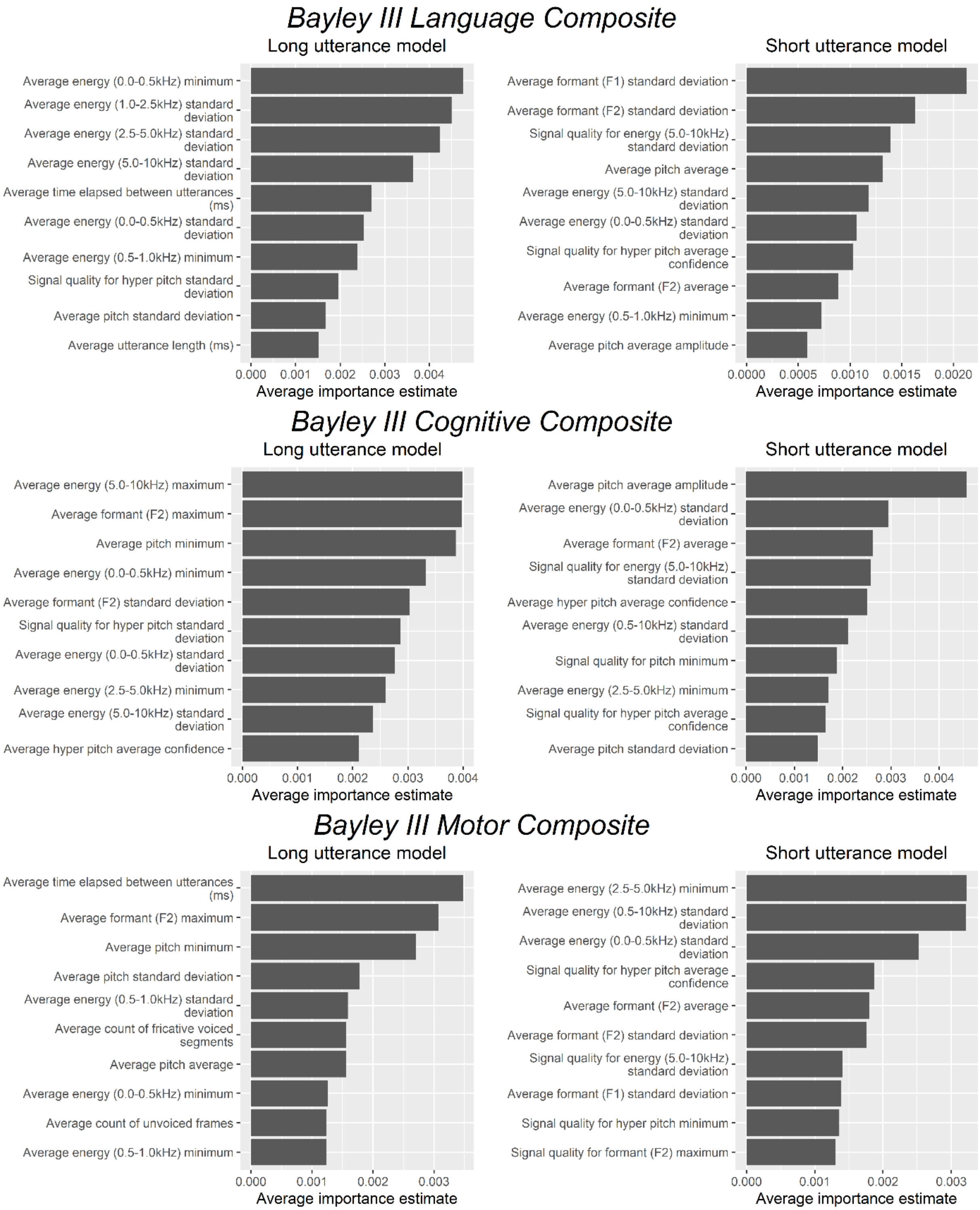

eFigure 2. Feature Importance Estimates From Models Predicting Bayley-III Composite Scores Using Cry Acoustic Characteristics (Only the Top 10 Features are Shown)

© 2023 Manigault AW et al. *JAMA Network Open.*

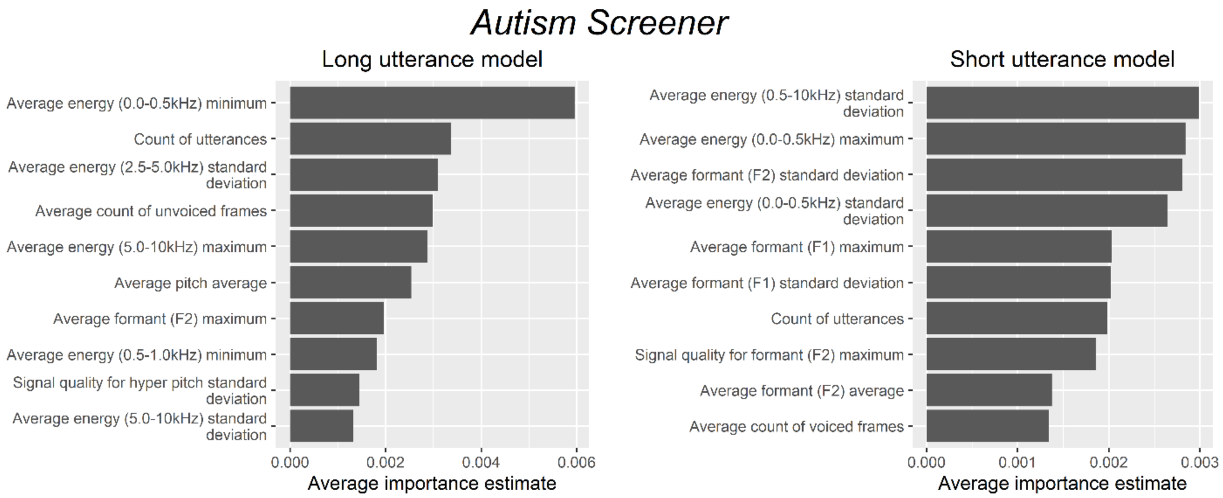

eFigure 3. Feature Importance Estimates From Models Predicting MCHAT Positive Autism Screen Using Cry Acoustic Characteristics (Only the Top 10 Features are Shown)

© 2023 Manigault AW et al. *JAMA Network Open.*
