# Supplementary material for: Acoustic Cry Characteristics in Preterm Infants and Developmental and Behavioral Outcomes at 2 Years of Age
Source: JAMA Netw Open. 2023 Feb 1;6(2):e2254151. doi: 10.1001/jamanetworkopen.2022.54151 (PMC9892956; doi:10.1001/jamanetworkopen.2022.54151)
Supplement: Supplement 2. — Data Sharing Statement [file jamanetwopen-e2254151-s002.pdf]

Data Sharing Statement

Manigault. Acoustic Cry Characteristics in Preterm Infants and Developmental and Behavioral Outcomes at 2 Years of Age. *JAMA Netw Open*. Published February 01, 2023. doi:10.1001/jamanetworkopen.2022.54151

Data
Data available: No
